# Building the drug-GO function network to screen significant candidate drugs for myasthenia gravis

**DOI:** 10.1371/journal.pone.0214857

**Published:** 2019-04-04

**Authors:** Shuang Li, Yuze Cao, Lei Li, Huixue Zhang, Xiaoyu Lu, Chunrui Bo, Xiaotong Kong, Zhaojun Liu, Lixia Chen, Peifang Liu, Yang Jiao, Jianjian Wang, Shangwei Ning, Lihua Wang

**Affiliations:** 1 Department of Neurology, The Second Affiliated Hospital, Harbin Medical University, Harbin, Heilongjiang Province, China; 2 Department of Neurology, Peking Union Medical College Hospital, Chinese Academy of Medical Sciences, Beijing, China; 3 College of Bioinformatics Science and Technology, Harbin Medical University, Harbin, Heilongjiang Province, China; Chuo University, JAPAN

## Abstract

Myasthenia gravis (MG) is an autoimmune disease. In recent years, considerable evidence has indicated that Gene Ontology (GO) functions, especially GO-biological processes, have important effects on the mechanisms and treatments of different diseases. However, the roles of GO functions in the pathogenesis and treatment of MG have not been well studied. This study aimed to uncover the potential important roles of risk-related GO functions and to screen significant candidate drugs related to GO functions for MG. Based on MG risk genes, 238 risk GO functions and 42 drugs were identified. Through constructing a GO function network, we discovered that positive regulation of NF-kappaB transcription factor activity (GO:0051092) may be one of the most important GO functions in the mechanism of MG. Furthermore, we built a drug-GO function network to help evaluate the latent relationship between drugs and GO functions. According to the drug-GO function network, 5 candidate drugs showing promise for treating MG were identified. Indeed, 2 out of 5 candidate drugs have been investigated to treat MG. Through functional enrichment analysis, we found that the mechanisms between 5 candidate drugs and associated GO functions may involve two vital pathways, specifically hsa05332 (graft-versus-host disease) and hsa04940 (type I diabetes mellitus). More interestingly, most of the processes in these two pathways were consistent. Our study will not only reveal a new perspective on the mechanisms and novel treatment strategies of MG, but also will provide strong support for research on GO functions.

## Introduction

Myasthenia gravis (MG) is an autoimmune disease of chronic neuromuscular disorder mainly caused by the antibodies against nicotinic acetylcholine receptor (AChR) in the postsynaptic membrane [1]. The primary clinical manifestations of MG include fluctuating muscle weakness and fatigue, which can range from mild forms affecting only the eye muscles to severe generalized forms. Many studies have elucidated the pathogenesis of MG [2,3]. With improved diagnosis and prolonged survival, the prevalence of MG is growing in recent years [2,4]. However, the current treatment strategies have different degrees of side effects and none of them can completely cure MG.

In recent years, researching gene networks has become a focus. Vitali et al. constructed a protein-protein interaction (PPI) network to explore the genetic underpinnings of wound healing mechanisms [5]. Many researchers have also developed various algorithms to analyze or identify the network functions of genes or gene products, such as MTGO [6] and DCAFP [7], which provided great insight into the research of genes or gene products. Gene ontology (GO) project provides a set of comprehensive available resources on genes and gene products [8], which include concepts/classes to describe gene function and annotation. The project focuses on the following three aspects: molecular function (MF), cellular component (CC) and biological process (BP). In recent years, GO-biological process (GO-BP) has been the focus of multiple research projects. For example, while exploring autophagy with GO database, Paul et al. found that different types of autophagy require specific BP terms [9]. According to a novel form of network-based gene enrichment, Lena et al. proposed a more effective method for detecting BPs associated diseases [10], which may help us to better understand the mechanism of different diseases if we can determine the BPs of diseases. Another study has found that altered genes in bladder neoplasm patients were mainly enriched for two classes of BP through GO analysis, which suggests that these BPs may participate in the onset of this disease or worsen the observed phenotype [11]. In addition, Wirapati et al. discovered that the GO-BPs with high ‘coexpression’ genes could help to reveal the common thread connecting molecular subtyping and several prognostic signatures of breast cancer [12]. These studies indicated that GO-BP may have an important role in the initiation and progression of diseases. However, the potential role of GO-BP in MG is still unclear.

It has been reported that using old drugs for new indications has become an attractive form of drug discovery [13] that can save time and money compared to developing new drugs. For example, based on widely functions of miRNA, a miRNA-regulated drug-pathway network was constructed to recognize new treatment drugs for MG in our previous work [14,15]. However, a disease may be caused by many abnormally expressed genes, which in turn disturb the BPs that the genes participated. In addition, drugs can bind to target genes and influence the BPs in which the target genes are located. For instance, Lee et al. developed an efficient and useful way to investigate the relationships between BPs and side effects by building a process-drug-side effect network [16]. It seems that if we can determine abnormal BPs that are affected by differentially expressed genes, then some drugs that target those genes can be applied to offset the anomalies caused by the BPs. This approach will provide a new dimension for the treatment of diseases. Researchers have proposed a method for drug repositioning based on disease-associated GO-BP [17]. Meanwhile, Porrelloa and Piergentilib identified new potential therapeutic targets for bladder neoplasm by analyzing BP in which altered genes were enriched [11], which provided strong support for screening new drugs based on GO-BP. However, no studies have focused on the association between drugs and GO-BPs in MG.

**Fig 1:**
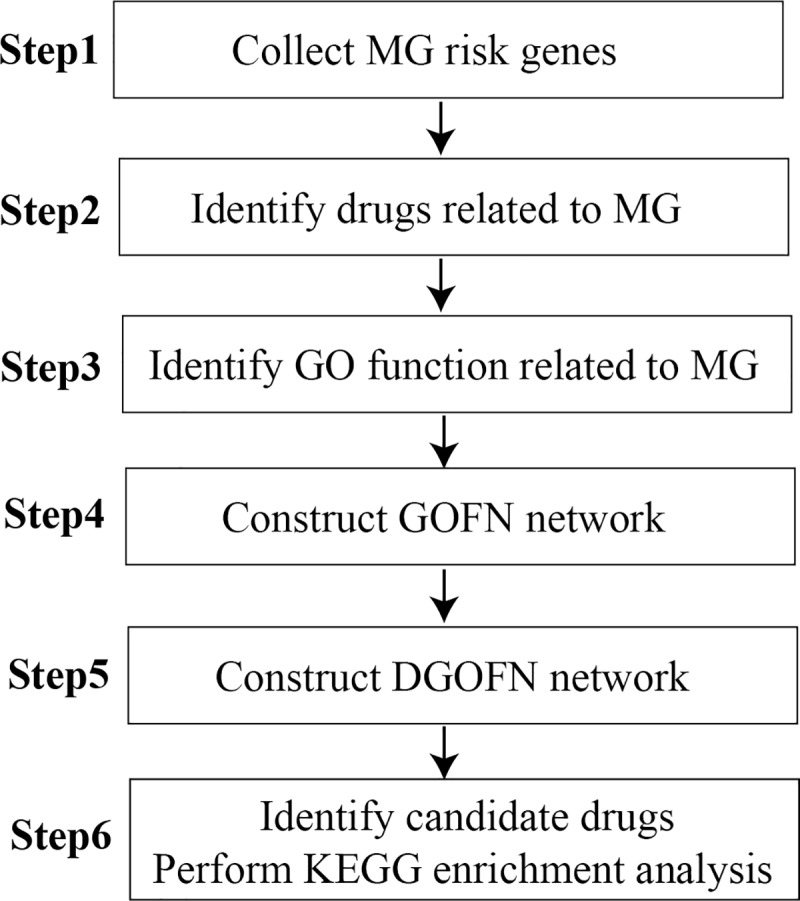
Flowchart of methods. Step 1: MG risk genes were collected from current public databases. Step 2: According to the cumulative hypergeometric distribution, we identified statistically significant GO functions for MG. Step 3: Based on MG risk genes, we acquired drugs related to MG. Step 4: Based on the cumulative hypergeometric formula, we obtained statistically significant GO function pairs and constructed a GOFN network. Step 5: According to the cumulative hypergeometric formula, we obtained statistically significant drug-GO function pairs and constructed a DGOFN network. Step 6: According to the DGOFN, we calculated the association scores (AS) and the specificity of the association and dissected the mechanism between candidate drugs and GO functions in pathways.

In this study, as shown in the flowchart (Fig 1), we identified risk-related GO functions and recognized drugs based on MG risk genes. Then, we constructed GO function network (GOFN) and drug-GO function network (DGOFN). We found an important immune-related GO function and revealed several new drug candidates for MG by calculating Z-value between drugs and MG. Finally, we identified two risk pathways regulated by MG risk genes and drugs, which might interact with GO functions. These results may provide potential guidance for identifying the mechanisms and treatments for MG.

## Data sources

### Data for human MG risk genes

MG risk gene data were collected in two ways. For one thing, we manually browsed 12,454 items by searching literature published before March 1st, 2018 in PubMed database using the terms [myasthenia gravis (MeSH Terms) and English (Language)], and then we selected eligible genes. The selection criteria for an MG risk gene was consistent with our previous studies published online [18]: the risk gene was significantly differently expressed in at least 5 MG samples (including blood samples or thymic samples) and was detected using dependable experimental methods (such as ELISA, RT-PCR and Western Blot). For another thing, we also obtained MG risk gene data from three current public databases, including Online Mendelian Inheritance in Man (OMIM) (March 1st, 2017, https://omim.org) [19], the Genetic Association Database (GAD) (September 1st, 2014, http://geneticassociationdb.nih.gov) [20] and DisGeNET (version 5.0, http://www.disgenet.org) [21]. Finally, we collected 258 MG risk genes, including 144 risk genes obtained through a literature search (detailed information in S1 Table) and 114 risk genes compiled from public databases (shown in S2 Table)

### Human gene ontology data

The information for GO annotation was download from GO database (http://www.geneontology.org), with the species restricted to “*Homo sapiens*”. Based on the genes we collected, we identified MG related GO functions in GO level 3 (we selected GO-BPs to represent GO functions) [10,14]. The GO functions with less than 5 MG risk genes were excluded from this study.

### Drugs and drug targets data

The data for drugs and their target genes were downloaded from DrugBank database (version 5.1.1) [22]. The species was limited to “*Homo sapiens*”. By intersecting MG risk genes we collected with drug targets, we obtained 464 drugs that might target MG. Then, we excluded drugs with less than 3 target genes from this study. Finally, we obtained 43 drugs that targeted MG risk genes.

## Methods

### Human MG risk gene ontology annotation

We performed an enrichment analysis to annotate human MG risk genes using the following formula:

$$P=F(x|M,K,N)=\sum_{i=0}^{x}\frac{\left(\begin{array}{c} K\\ i \end{array}\right)\left(\begin{array}{c} M-K\\ N-i \end{array}\right)}{\left(\begin{array}{c} M\\ N \end{array}\right)}$$ (1)

At first, the enrichment analysis was applied for selecting GO functions significantly related to MG. Using this approach, M denoted the total number for the human whole genome, K denoted the number of genes in a given GO function, N denoted the total number of MG risk genes, and x represented the number of overlapping genes between GO function and MG risk genes. Statistical significance was achieved if the P-value was less than 0.05.

### Construction of networks

The enrichment analysis (the formula (1)) was also performed in the construction of networks, including GO function network (GOFN) and drug-GO function network (DGOFN). First, when analyzing every two random GO function pairs, M represented the total number of the human whole genome, N represented the total number of genes in one GO function, K denoted the number of genes in another GO function, and x was the number of overlapping genes between two GO functions. The association between each GO function and all other GO functions was analyzed. Similarly, for each drug-GO function pair, M denoted the total number of human MG risk genes, K denoted the number of genes in a given GO function, N denoted the number of target genes of a given drug, and x represented the number of overlapping genes between GO functions and drugs. After calculating the p-value between every random GO function pair or drug-GO function pair, we adjusted the p-value using the Benjamini and Hochberg false discovery rate (FDR) to determine statistical significance. We considered a GO function pair and a drug-GO function pair to be notably overlapping if the FDR was less than 0.05 and constructed separately GOFN and DGOFN networks. Next, Cytoscape 3.6.0 was used to visualize the networks.

### Functional enrichment analysis in pathways

We carried out KEGG pathway enrichment analysis to identify MG risk pathways that included significant candidate drug targets (MG risk genes) that were enriched using the functional annotation tools in DAVID [23]. We defined an FDR value less than 0.05 as the cutoff.

### Calculation of degree and betweenness

In the GOFN, the degree of a GO function was the number of the other GO functions which were connected to the GO function. Similarly, the degree of a GO function (or a drug) was the number of drugs (or GO functions), which were connected to the GO function (or the drug) in the DGOFN. For a GO function ‘v’ in GOFN, the betweenness of ‘v’ was the sum of the numbers of the shortest paths between all pairs of GO functions through the node ‘v’. In this study, we calculated the node of betweenness by using the package igraph for R and the Network Analysis plugin [24] of Cytoscape was used to analyze the network properties.

### Association scores and screening significant drugs for MG

Based on the DGOFN, we calculated the association scores (AS) and the specificity of the association by using the formulas from our previous study [14]. The formulas were as follows:

$$S_{drugi,MG}=-\mathrm{lg}\sum \sqrt{P_{drugi,k}\times P_{MG,k}}$$ (2)

$$Z_{drugi,MG}=\frac{S_{drugi,k}-average(S_{random,drugi})}{std(S_{random,drugi})}$$ (3)

In formula (2), P_drugi,k_ was the P-value of drug ‘i’ enriched in GO function ‘k’; P_MG,k_ was the P-value of MG enriched in GO function ‘k’; and ‘k’ was used to represent the most significant GO function affected by drug ‘i’ targets and MG risk genes. We obtained the S value of each candidate drug after applying formula (2). Next, to assess the specificity of the association between drugs and MG, we conducted a permutation of the GO functions and computed the Z scores of the drugs and MG by using formula (3). We also obtained the random GO function profiles of the drugs by randomly ranking the GO function 10,000 times. For each random profile, S_random,drugi_ was calculated according to formula (2); the average(S_random,drugi_) represents the average association score between random cases and drug ‘i’; and std(S_random,drugi_) represents the standard variation of association between random cases and drug ‘i’. Z_drugi,MG_ was the significant score between drug ‘i’ and MG. The higher the Z-value, the more significant the association between drug and MG was. The drugs can be regarded as candidates for MG treatment if the Z-value >1.96 (P<0.05).

### Validation using GEO dataset

The human microarray dataset GSE85452 [25] was downloaded from the NCBI Gene Expression Omnibus (www.ncbi.nlm.nih.gov/geo/). The GSE85452 microarray dataset was generated with the GPL10558 platform (Illumina HumanHT-12 V4.0 expression beadchip) and included 13 MG patients and 12 controls. T-test was applied to identify the differential expressed genes (P < 0.05, |fold change (FC)| >2). According to a hypergeometric test, the overlap of MG risk genes and differential expressed genes in GSE85452 data were statistically significant if the P-value was less than 0.05.

## Results

### Identification of MG-related GO functions

**Fig 2:**
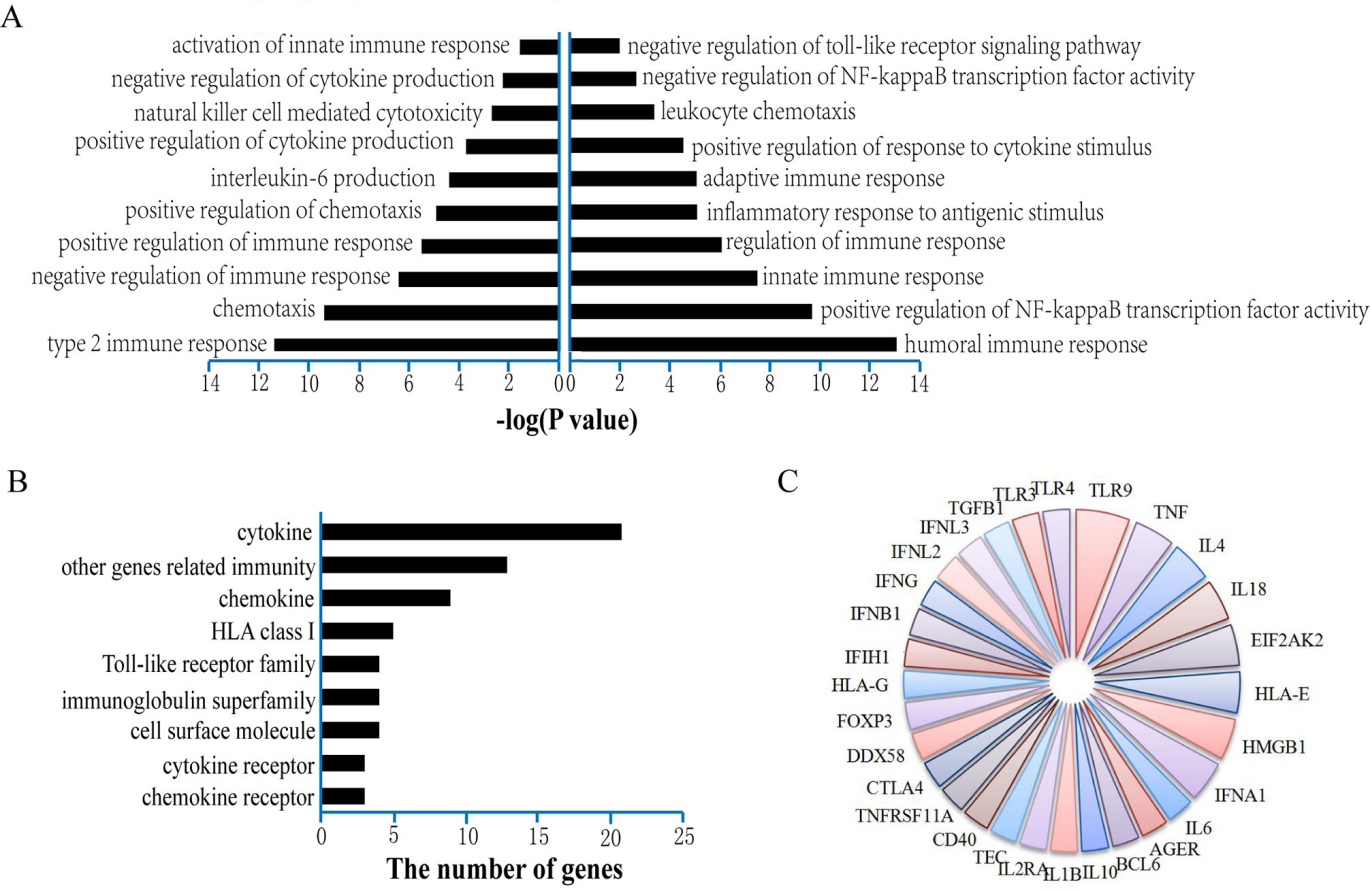
Twenty GO functions of myasthenia gravis. Twenty immune-related GO functions enriched by MG risk genes (P<0.05). (B) Functional classification of MG risk genes in 20 immune-related GO functions. (C) The frequency of MG risk genes in 20 immune-related GO functions.

A catalog of 258 risk genes was created. In terms of these MG risk genes, 238 risk GO functions (P<0.05) were identified (the detailed information was summarized in S3 Table). In this study, we mainly concentrated on immune-related GO functions due to the complex immunological mechanism of MG [26]. A total of 20 immune-related GO functions (P<0.05) were observed manually and were shown in Fig 2A. Other types of GO functions, such as cell development, defense response and tissue homeostasis, were shown in S3 Table. MG has been reported as a humoral immunity-mediated autoimmune disease. Epstein-Barr virus (EBV) infection was observed in B cells and plasma cells (PCs) in the thymus of patients with MG, which provided a possible theoretical basis for the mechanism through which humoral innate immunity induces autoimmunity in MG [26,27]. In addition, increasing evidence indicated the modulation of immune response and the presence of inflammation could contribute to MG mechanism [28,29] which have shown that these GO functions might exert potential important effects in the pathogenesis of MG.

Sixty-six MG risk genes were included in the 20 immune-related GO functions. According to the functional description of MG risk genes enriched in 20 immune-related GO functions, these genes could be predominantly grouped into 9 categories (Fig 2B). We found that most of the MG risk genes belonged to cytokines. It has been reported that cytokines were likely to have major importance in the pathogenesis of MG [30]. At the same time, we also analyzed MG risk genes located in 20 immune-related GO functions in a PPI network and obtained a subnetwork (S1 Fig). The degree distribution of nodes in the subnetwork was displayed in S2 Fig. We found that *TGFB1* with the highest degree was part of cytokines in Fig 2B, which suggested that MG risk genes played an important role in the PPI network and GO functions. The risk genes in the 20 immune-related GO functions which appeared more than once were shown in Fig 2C. The more frequently a gene appeared, the more widely it participated in GO functions, such as Toll-like receptor 9 (*TLR9*) and tumor necrosis factor (*TNF*). For example, Cavalcante et al. discovered that *TLR9* affected by EBV might cause the onset or maintenance of the autoimmune response in the intrathymic pathogenesis of MG [31]. These results suggest that the GO functions we identified may be crucial components of the mechanism of MG and provide a new direction for studying the pathogenesis of MG.

### Construction of GO function network and analysis of the network properties

**Fig 3:**
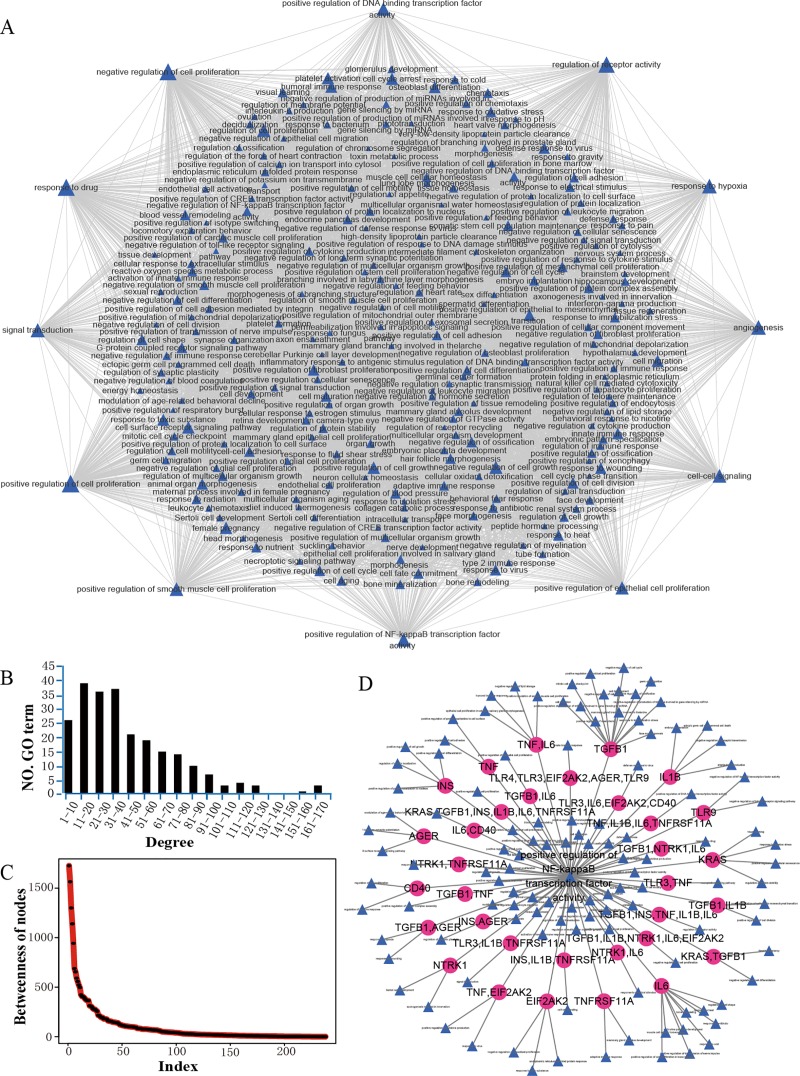
The associations among GO functions. GO function network (GOFN) in MG. Blue triangles represent GO functions; the size of the node represents the magnitude of the degree; the edge represents the connection between the two GO functions. (B) Degree distribution of all the nodes in the GOFN. (C) Node betweenness in the GOFN. (D) The dissection between the GO function for the positive regulation of NF-kappaB transcription factor activity and its connected GO functions. Blue triangles represent GO functions; purple circles represent MG risk genes; the interaction of two GO functions via MG risk genes is shown in the figure.

Based on the links of the 238 GO functions, we constructed a GOFN (Fig 3A). The network contained 238 nodes and 5167 interactions.

We analyzed the topological characteristics of the network. First, the degrees of the nodes in the GOFN were shown in Fig 3B. A small number of GO functions had high connectivity, such as the GO term of positive regulation of NF-kappaB transcription factor activity (GO:0051092), which had the highest degree among the immune-related GO functions and meant that it was highly relevant to the other GO functions it was connected to. Meanwhile, we also calculated the betweenness of the nodes in GOFN (Fig 3C). The higher the betweenness of the node, the more important this node was in maintaining tight connectivity in the network. Similarly, the GO term of GO:0051092 had the highest betweenness in all of the immune-related GO functions, which illustrated this GO term could have a wide range of functions. It has been found that GO:0051092 might play a core role in the GOFN network based on the analysis of the network properties, therefore, to figure out the association between the GO term of GO:0051092 and the other GO functions, a subnetwork was determined (Fig 3D) by dissecting the GO term of GO:0051092 in depth. As shown, the term was connected to 117 GO functions, and 79 of these GO functions were connected through MG risk genes, which contained 15 MG risk genes, including *TRL9*, *TNF*, *EIF2AK2*, *IL6*, *AGER*, *IL1B*, *CD40*, *TNFRSF11A*, *TGFB1*, *TLR3*, *TLR4*, *KRAS*, *INS* and *NTRK1*. It has been reported that NF-kappaB transcription factor can mediate inducible expression of several genes involved in inflammatory immune responses and many other BPs, which makes it a critical regulator of the inflammatory immune response [32,33]. Our results were consistent with those of previous studies.

### Construction of drug-GO function network and analysis of its topological features

**Fig 4:**
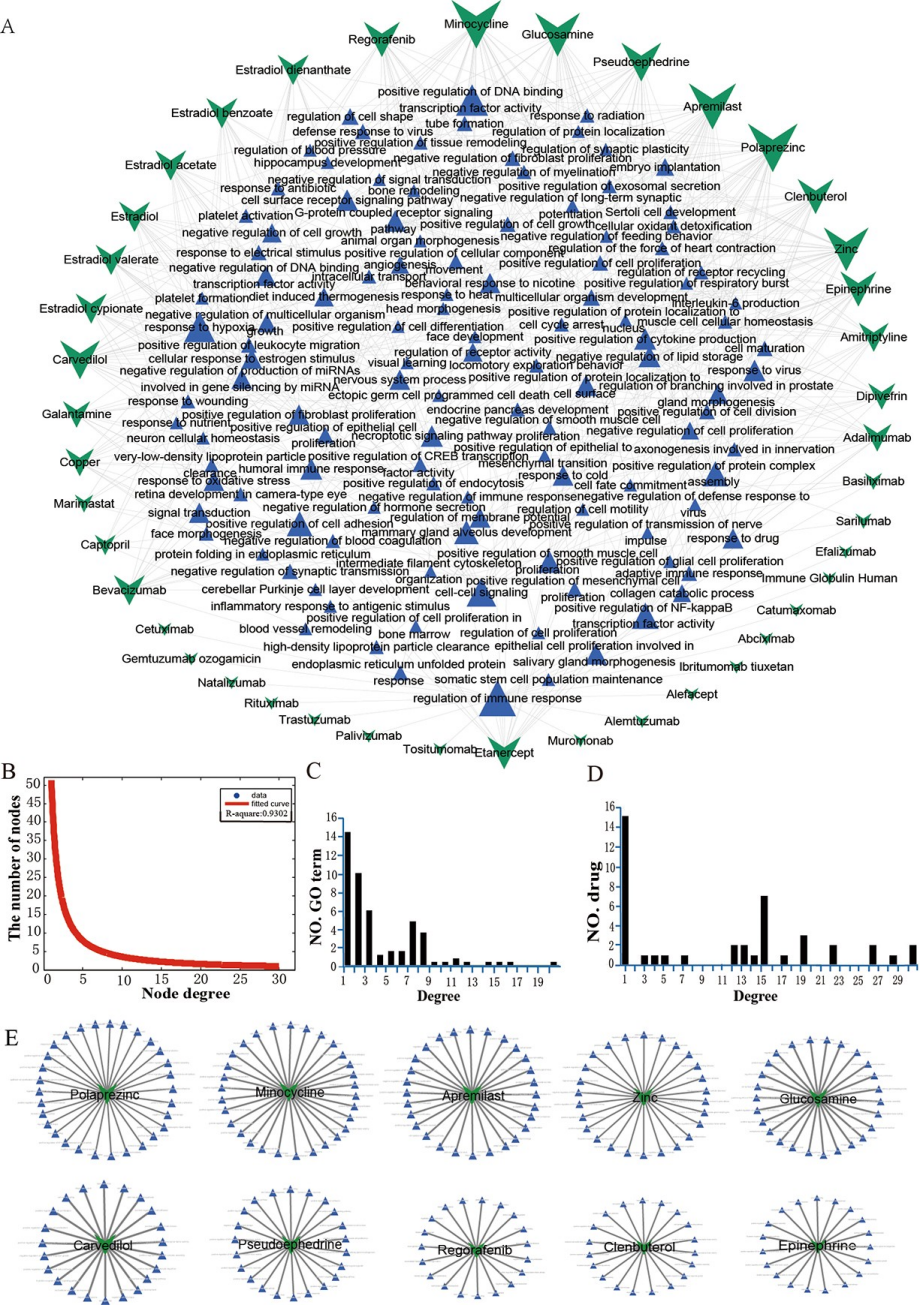
The relationship between drugs and GO functions. (A) Drug-GO function network (DGOFN) in MG. Network organization of drug and GO function associations. Green ‘V’ formations represent drugs; blue triangles represent GO functions. The size of the node represents the size of the degree; edge represents the connection between drugs and GO functions. (B) Degree distribution for all nodes in the DGOFN. (C) Degree distribution for the GO function nodes. (D) Degree distribution for the GO nodes. (E) The top 10 drugs ranked by drug degree (Polaprezinc, Minocycline, Apremilast, Zinc, Glucosamine, Carvedilol, Pseudoephedrine, Regorafenib, Clenbuterol and Epinephrine).

To understand how drugs (data sources) affect the GO functions, we identified 461 drug-GO function pairs (FDR value<0.05) and established the DGOFN (Fig 4A). According to the statistical analysis, 37 MG risk genes were enriched in all the drug-GO function pairs (not shown in the DGOFN). Meanwhile, 461 significant drug-GO function pairs between 42 drugs and 117 GO functions, which were from 238 risk GO functions, were contained in the network.

To comprehend the topological features of the DGOFN in detail, we characterized its degree distribution. The degree distribution of all the nodes followed a power law distribution *f*(*x*) = 51.28*x*^−1.16^ in the DGOFN (Fig 4B). We also determined the degree distribution of GO functions and drugs in the DGOFN. The degree distribution of GO functions was displayed in Fig 4C, the degree of regulation of immune response (GO:0050776) was 20, which was the highest degree in all GO functions, i.e., 20 drugs could act on this GO function. In addition, the GO term of GO:0051092 had the second highest degree of all the immune-related GO functions. These results indicated that immune-related GO functions may be important potential therapeutic targets for MG. The degree distribution of drugs was shown in Fig 4D, which suggested that most of the drugs can influence more than one GO function. Furthermore, we demonstrated the top 10 drugs and the GO functions associated with these drugs in Fig 4E, which might provide more options for the target therapy of MG.

### MG candidate drugs and associated GO functions

**Fig 5:**
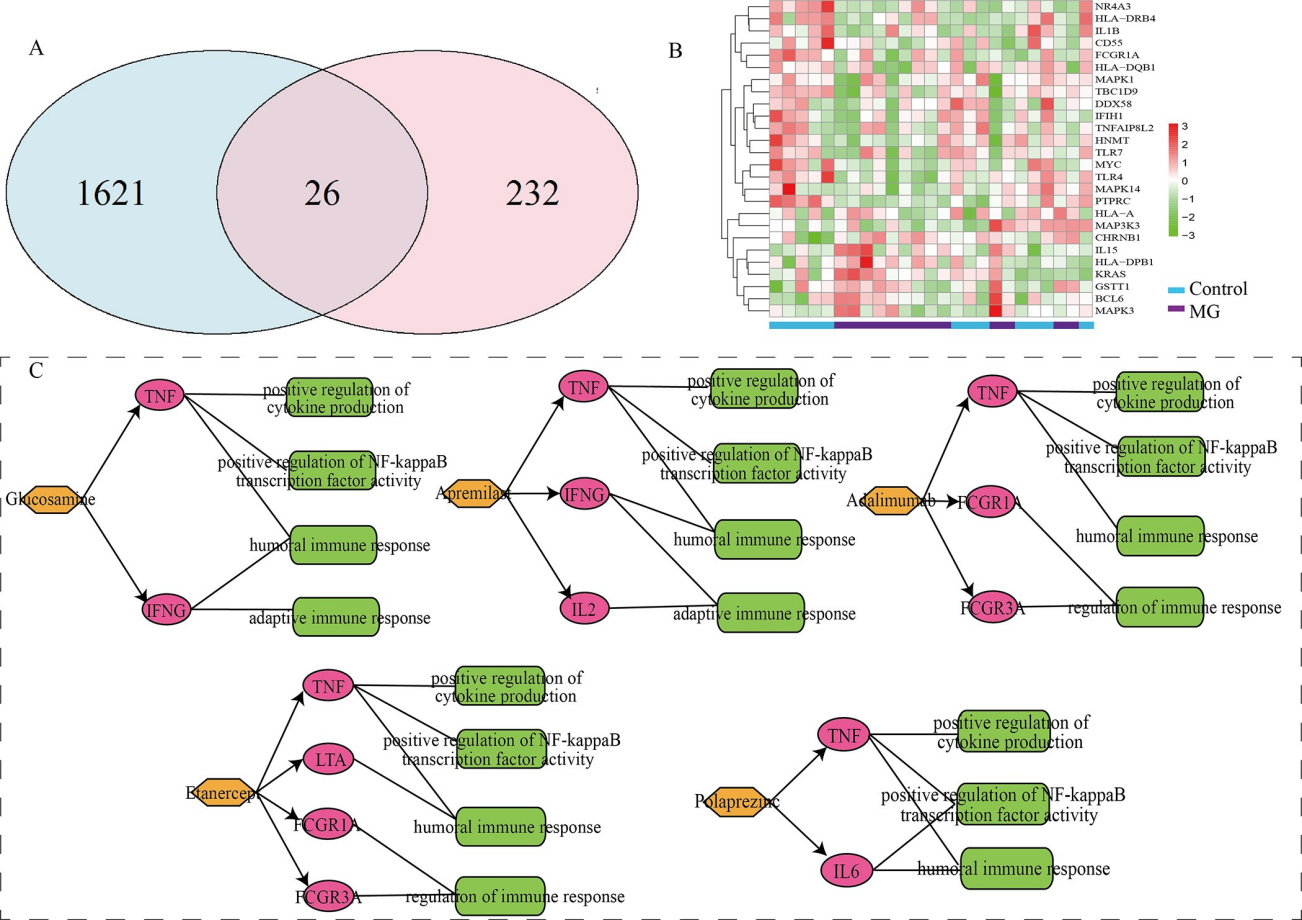
Venn diagram, heatmap and Layered networks. A. Venn diagram of MG risk genes and differentially expressed MG genes; light blue ellipse indicates differentially expressed MG genes, pink ellipse indicates MG risk genes; purple intersection indicates overlapping genes. B. Heatmap of overlap between MG risk genes and differentially expressed MG genes; blue rectangles represent control samples, purple rectangles represent MG samples. C. Layered networks, including Glucosamine-GO functions network, Apremilast-GO functions network, Adalimumab-GO functions network, Etanercept-GO functions network and Polaprezinc-GO functions network; green round rectangles indicate GO functions; purple ellipses indicate MG risk genes; orange hexagons indicate candidate drugs.

According to the DGOFN, we calculated Z scores of drugs and MG (methods). Five candidate drugs with Z-value >1.96 (P<0.05) were identified, including Glucosamine, Apremilast, Adalimumab, Etanercept and Polaprezinc. To verify the reliability of our results, we downloaded the gene expression profile of MG (GSE85452) from Gene Expression Omnibus (GEO) database (http://www.ncbi.nlm.nih.gov/geo/). By analyzing high-throughput expression profile, we found that 1647 MG genes were differentially expressed (Fig 5A). The overlap of MG risk genes and differentially expressed MG genes was statistically significant (p<0.05) based on a hypergeometric test (Fig 5B). Importantly, MG risk gene-*FCGR1A*, which is the target of Adalimumab and Etanercept, was shown to be differentially expressed in the expression profile of GSE85452. In fact, Adalimumab and Etanercept have been investigated to treat MG. These findings further enhanced that our results were reliable.

In principle, the same drug can often be used to treat other diseases that share the affected BPs. Therefore, we also confirmed the reliability of our candidate drugs by consulting the relevant literature in PubMed. Glucosamine, which is a common dietary supplement, has immunosuppressive effects on autoimmune diseases [34,35]. Chien et al. confirmed Glucosamine could reduce *IL2* downstream signaling through downregulating *IL2RA* [36], while *IL2* and *IL2RA* (*CD25*) were upregulated in patients with MG, and *IL2RA* might affect the clinical symptoms of MG [37,38]. These results show that Glucosamine has the potential to be a therapeutic target for MG. Apremilast is a novel inhibitor of phosphodiesterase 4 that has led to great interest in targeted treatments for autoimmune diseases [39] It was reported that Apremilast has been approved for psoriasis and psoriatic arthritis [40]. Furthermore, Apremilast can inhibit the generation of cytokines such as *TNF*, *IL-2*, *CXCL10* and *CCL4* [41], which are all MG risk genes. These studies suggest that Apremilast is promising for treating MG.

Next, to more intuitively illustrate the relationship among MG risk genes, 5 candidate drugs and the associated GO functions, we built drug-GO function layered networks. We divided the GO functions associated with drugs into immune-related functions and immune-unrelated functions. Then, we selected the immune-related GO functions to build layered networks (Fig 5C). Researchers have concluded that targeting *NF-kappaB* as well as its related signaling pathways could be a potential therapeutic target for cancer treatments [42]. However, MG is mainly caused by thymoma and may also be related to other kind of cancers, which implies that GO functions may be key factors for the treatment of MG. We can see that GO term of GO:0051092 was associated with all of the candidate drugs through *TNF*. A study showed that *TNF* was one of the most important cytokines in the mechanism of MG, and inhibiting *TNF* may exert notable clinical efficacy for MG [43], which indicates that *TNF* may be a significant therapeutic target in the future. We summarized the information on target genes among the 5 candidate drugs and immune-unrelated GO functions in S4 Table.

### Mechanism dissection of MG candidate drugs and associated GO functions in pathways

**Fig 6:**
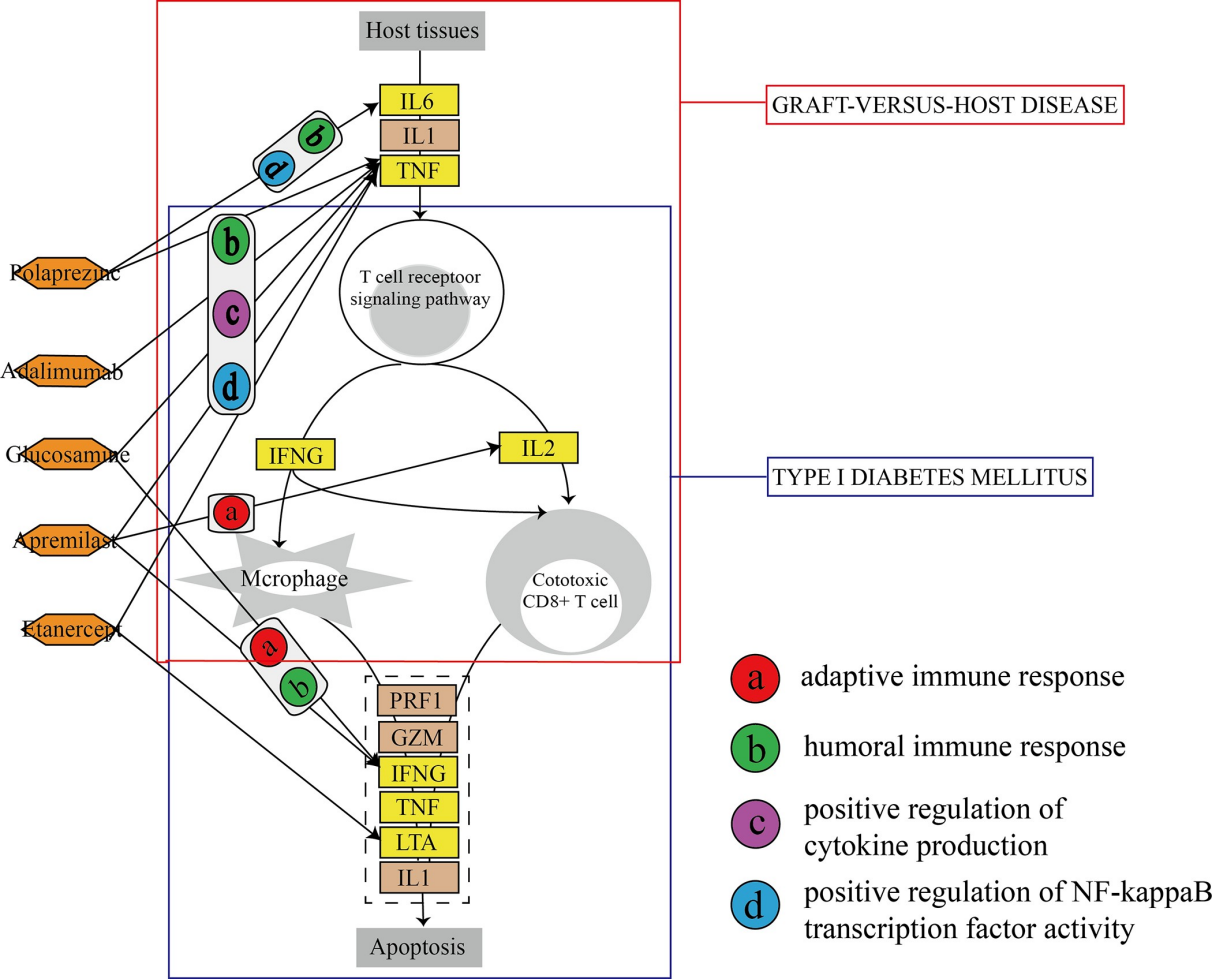
Dissection of mechanism between candidate drugs and GO functions in pathways. The rectangle with the red lines indicates hsa05332 (graft-versus-host disease); the rectangle with the blue lines indicates hsa04940 (type I diabetes mellitus). Yellow rectangles indicate MG risk genes; orange hexagons indicate drugs; circles indicate GO functions.

Finally, to investigate the underlying mechanisms between the 5 candidate drugs and the GO functions affected by these candidate drugs in pathways, we performed KEGG pathway enrichment analysis based on the target genes between the 5 candidate drugs and GO functions. As a result, we identified 37 MG risk pathways (FDR value<0.05). Among these pathways, we discovered that hsa05332 (graft-versus-host disease) and hsa04940 (type I diabetes mellitus) were the two most statistically significant pathways (Fig 6). Myasthenic symptoms are frequently associated with other symptoms of chronic graft-versus-host disease (GVHD) and MG has been reported as a rare complication of chronic GVHD after allogeneic hematopoietic stem cell transplantation [44–46]. Additionally, it has been evidenced that the development of type 1 diabetes increases the risk of other autoimmune diseases and is related to genetic susceptibility for development of these diseases [47]. Consequently, these two pathways are closely related to the pathogenesis of MG. Forty-two GO functions were involved in the hsa05332 pathway, whereas 31 GO functions were associated with the hsa04940 pathway. Immune-related GO functions participating in the two pathways were displayed in Fig 6 (the remaining GO functions were shown in S4 Table). As we can see, *TNF*, *IL2* and *INFG* are the common genes in both pathways. Because the two pathways were regulated by the most of the same risk genes, the candidate drugs and associated GO functions were almost similar. These results demonstrate that the GO functions we identified are useful for discovering candidate drugs and are extremely crucial in the mechanism of MG. Meanwhile, our results will provide a new direction for clarifying the pathogenesis and treatments of MG.

## Discussion

In this study, we have identified the potential mechanism of risk GO functions based on the current knowledge of MG and screened significant candidate drugs for MG for the first time. Through compiling the MG risk gene catalog, we enriched MG risk GO functions. Furthermore, we constructed the GOFN and demonstrated the importance of GO functions; we also built the DGOFN and revealed candidate drugs that may affect risk-related GO functions. Finally, we performed an enrichment analysis in pathways and proposed a potential mechanism between GO functions and drugs.

The GO functions we identified reveal an overview of MG pathogenesis. They also provide strong support for further investigation of GO functions. MG is an autoimmune disease, immune-related GO functions may be more closed to the pathogenesis of MG. In the study, we mainly focused on 20 immune-related GO functions. It has been reported that immune response played important roles in the mechanism of MG [48,49], which provide the basis for our study. Several significant GO functions associated with MG were discovered, which may contribute to the onset of MG. NF-kappaB was induced during B cells maturation, therefore, it may have a major role in the activation and the development of B cells [50] while B cells are critical contributors to the humoral immune response and can produce various antibodies. In addition, NF-kappaB can activate cells of the innate immune system when inflammation occurs [51]. Once NF-kappaB is triggered, a series of inflammatory immune responses may occur that may accompany the production of antibodies, including AChR antibody and thus induce MG. In addition, the classification of MG risk genes in immune-related GO functions further highlighted the fundamental characteristics of autoimmune MG.

The GOFN network demonstrated that all of the GO functions were significantly correlated with some of the remaining GO functions. According to the topological properties of GOFN, we further implicated the potential significant role of GO:0051092 in MG. NF-kappaB is part of a complex family of proteins that not only can mediate many crucial biological functions through innate and adaptive immunity [50,52], but can also influence the expression of genes participating in inflammatory immune responses. For example, miR-26 can downregulate *IL6* production through silencing the expression of *MALT1* and *HMGA1*, while *MALT1* and *HMGA1* are two proteins with vital functions in *NF-kappaB* [53]. By dissecting GO:0051092 in depth, we discovered that this GO term did interact with other GO functions through some important MG risk genes. Therefore, we speculated that GO:0051092 was one of the most important GO functions in MG.

While building the GOFN network, we also built the DGOFN network to identify significant candidate drugs related to GO functions. The significant candidate drugs we acquired were related to MG or other autoimmune diseases, supported by related literature. The effects of such potential discoveries are broad because they might lead to accurate targeted therapeutics and individual treatments. Our previous study considered the pathways that were enriched by MG risk genes and miRNAs [14], whereas we mainly focused on the GO functions related MG to identify MG candidate drugs for this study. Starting from a different perspective, we again recognized several significant candidate drugs for MG. Our results further illuminate that GO functions may have more prospects for research on MG pathogenesis and for screening candidate drugs for diseases. However, well-designed experiments are still essential to confirm whether these drugs can be used to treat MG. Nonetheless, our research will serve as an important complement to future experimental studies of GO functions and drugs in MG, particularly because of the lack of exploration in this field to date.

In conclusion, we compiled a catalog of MG risk genes and identified risk GO functions, drugs and risk pathways. We constructed a GOFN to help understand the association between GO functions. We also investigated the complex connection among MG risk genes, drugs and GO functions by constructing a DGOFN and we identified 5 candidate drugs. Furthermore, we dissected the regulatory mechanism of candidate drugs and associated GO functions in risk pathways. Our results may provide strong support and new viewpoint for further research on the mechanisms and treatments of MG.

## Supporting information

S1 Fig The PPI subnetwork of MG risk genes located in 20 immune-related GO functions; blue circles represents genes. (TIF)

S2 Fig The degree distribution of nodes in the PPI subnetwork. (TIF)

S1 Table MG risk genes obtained from a literature search. (DOC)

S2 Table MG risk genes downloaded from three current databases. (DOC)

S3 Table Detailed information on 238 GO functions. (XLS)

S4 Table Target genes between 5 candidate drugs and GO functions. (DOC)
