# Supplementary figures and images for: Building the drug-GO function network to screen significant candidate drugs for myasthenia gravis
Source: PLoS One. 2019 Apr 4;14(4):e0214857. doi: 10.1371/journal.pone.0214857 (PMC6448860; doi:10.1371/journal.pone.0214857)

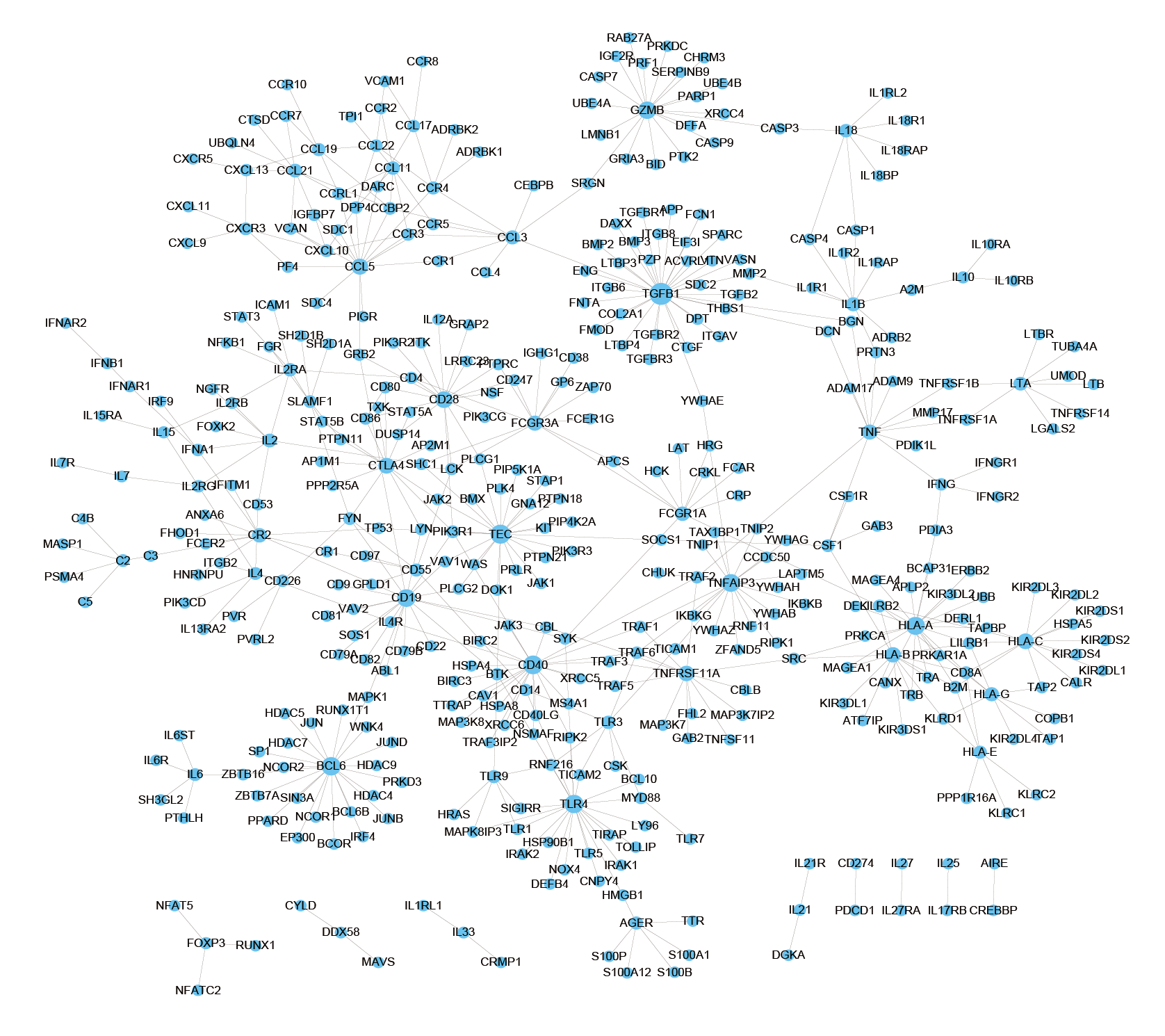

Supplement: S1 Fig — (TIF) [file pone.0214857.s001.tif]

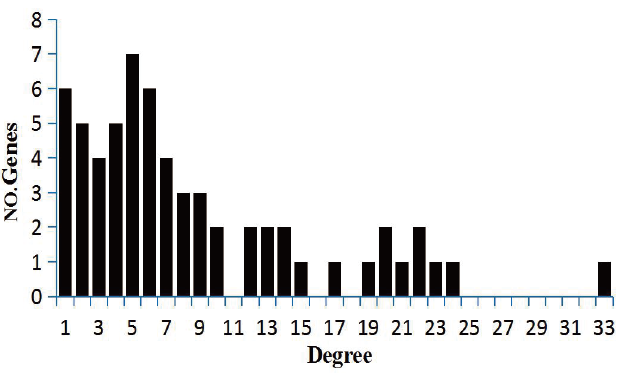

Supplement: S2 Fig — (TIF) [file pone.0214857.s002.tif]
