# Supplementary material for: Building the drug-GO function network to screen significant candidate drugs for myasthenia gravis
Source: PLoS One. 2019 Apr 4;14(4):e0214857. doi: 10.1371/journal.pone.0214857 (PMC6448860; doi:10.1371/journal.pone.0214857)
Supplement: S2 Table — (DOC) [file pone.0214857.s004.doc]

Table S2. MG risk genes downloaded from three current databases.

| **Disease Name** | **Gene** | **Symbol** | **Gene Name** | **# Of PMIDs** |
| --- | --- | --- | --- | --- |
| Myasthenia Gravis | 4593 | MUSK | muscle, skeletal, receptor tyrosine kinase | 5 |
| Myasthenia Gravis | 3115 | HLA-DPB1 | major histocompatibility complex, class II, DP beta 1 | 4 |
| Myasthenia Gravis | 629 | CFB | complement factor B | 1 |
| Myasthenia Gravis | 355 | FAS | Fas cell surface death receptor | 1 |
| Myasthenia Gravis | 5443 | POMC | proopiomelanocortin | 1 |
| Myasthenia Gravis | 4049 | LTA | lymphotoxin alpha | 5 |
| Myasthenia Gravis | 1515 | CTSV | cathepsin V | 1 |
| Myasthenia Gravis | 27335 | EIF3K | eukaryotic translation initiation factor 3, subunit K | 17 |
| Myasthenia Gravis | 7273 | TTN | titin | 12 |
| Myasthenia Gravis | 129831 | RBM45 | RNA binding motif protein 45 | 12 |
| Myasthenia Gravis | 5788 | PTPRC | protein tyrosine phosphatase, receptor type, C | 1 |
| Myasthenia Gravis | 3133 | HLA-E | major histocompatibility complex, class I, E | 11 |
| Myasthenia Gravis | 2100 | ESR2 | estrogen receptor 2 (ER beta) | 1 |
| Myasthenia Gravis | 5133 | PDCD1 | programmed cell death 1 | 1 |
| Myasthenia Gravis | 246750 | MYAS1 | Myasthenia gravis with thymus hyperplasia | 1 |
| Myasthenia Gravis | 940 | CD28 | CD28 molecule | 2 |
| Myasthenia Gravis | 5243 | ABCB1 | ATP-binding cassette, sub-family B (MDR/TAP), member 1 | 1 |
| Myasthenia Gravis | 2908 | NR3C1 | nuclear receptor subfamily 3, group C, member 1 (glucocorticoid receptor) | 1 |
| Myasthenia Gravis | 2214 | FCGR3A | Fc fragment of IgG, low affinity IIIa, receptor (CD16a) | 1 |
| Myasthenia Gravis | 1636 | ACE | angiotensin I converting enzyme | 1 |
| Myasthenia Gravis | 3669 | ISG20 | interferon stimulated exonuclease gene 20kDa | 7 |
| Myasthenia Gravis | 2651 | GCNT2 | glucosaminyl (N-acetyl) transferase 2, I-branching enzyme (I blood group) | 4 |
| Myasthenia Gravis | 146 | ADRA1D | adrenoceptor alpha 1D | 4 |
| Myasthenia Gravis | 3118 | HLA-DQA2 | major histocompatibility complex, class II, DQ alpha 2 | 3 |
| Myasthenia Gravis | 4155 | MBP | myelin basic protein | 3 |
| Myasthenia Gravis | 11319 | ECD | ecdysoneless homolog (Drosophila) | 3 |
| Myasthenia Gravis | 10699 | CORIN | corin, serine peptidase | 3 |
| Myasthenia Gravis | 7979 | SHFM1 | split hand/foot malformation (ectrodactyly) type 1 | 3 |
| Myasthenia Gravis | 6520 | SLC3A2 | solute carrier family 3 (amino acid transporter heavy chain), member 2 | 3 |
| Myasthenia Gravis | 8163 | CDR3 | Cerebellar degeneration-related autoantigen-3 | 3 |
| Myasthenia Gravis | 1E+08 | TEC | transient erythroblastopenia of childhood | 2 |
| Myasthenia Gravis | 266629 | SEC14L3 | SEC14-like 3 (S. cerevisiae) | 2 |
| Myasthenia Gravis | 79651 | RHBDF2 | rhomboid 5 homolog 2 (Drosophila) | 2 |
| Myasthenia Gravis | 27087 | B3GAT1 | beta-1,3-glucuronyltransferase 1 | 2 |
| Myasthenia Gravis | 8013 | NR4A3 | nuclear receptor subfamily 4, group A, member 3 | 2 |
| Myasthenia Gravis | 7006 | TEC | tec protein tyrosine kinase | 2 |
| Myasthenia Gravis | 3126 | HLA-DRB4 | major histocompatibility complex, class II, DR beta 4 | 2 |
| Myasthenia Gravis | 2209 | FCGR1A | Fc fragment of IgG, high affinity Ia, receptor (CD64) | 2 |
| Myasthenia Gravis | 958 | CD40 | CD40 molecule, TNF receptor superfamily member 5 | 2 |
| Myasthenia Gravis | 2261 | FGFR3 | fibroblast growth factor receptor 3 | 2 |
| Myasthenia Gravis | 590 | BCHE | butyrylcholinesterase | 2 |
| Myasthenia Gravis | 10717 | AP4B1 | adaptor-related protein complex 4, beta 1 subunit | 1 |
| Myasthenia Gravis | 836 | CASP3 | caspase 3, apoptosis-related cysteine peptidase | 1 |
| Myasthenia Gravis | 11200 | CHEK2 | checkpoint kinase 2 | 1 |
| Myasthenia Gravis | 799 | CALCR | calcitonin receptor | 1 |
| Myasthenia Gravis | 23158 | TBC1D9 | TBC1 domain family, member 9 (with GRAM domain) | 1 |
| Myasthenia Gravis | 859 | CAV3 | caveolin 3 | 1 |
| Myasthenia Gravis | 10598 | AHSA1 | AHA1, activator of heat shock 90kDa protein ATPase homolog 1 (yeast) | 1 |
| Myasthenia Gravis | 23586 | DDX58 | DEAD (Asp-Glu-Ala-Asp) box polypeptide 58 | 1 |
| Myasthenia Gravis | 10457 | GPNMB | glycoprotein (transmembrane) nmb | 1 |
| Myasthenia Gravis | 10381 | TUBB3 | tubulin, beta 3 class III | 1 |
| Myasthenia Gravis | 7432 | VIP | vasoactive intestinal peptide | 1 |
| Myasthenia Gravis | 7965 | AIMP2 | aminoacyl tRNA synthetase complex-interacting multifunctional protein 2 | 1 |
| Myasthenia Gravis | 1137 | CHRNA4 | cholinergic receptor, nicotinic, alpha 4 (neuronal) | 1 |
| Myasthenia Gravis | 1040 | CDS1 | CDP-diacylglycerol synthase (phosphatidate cytidylyltransferase) 1 | 1 |
| Myasthenia Gravis | 8548 | BLZF1 | basic leucine zipper nuclear factor 1 | 1 |
| Myasthenia Gravis | 1029 | CDKN2A | cyclin-dependent kinase inhibitor 2A | 1 |
| Myasthenia Gravis | 9260 | PDLIM7 | PDZ and LIM domain 7 (enigma) | 1 |
| Myasthenia Gravis | 9402 | GRAP2 | GRB2-related adaptor protein 2 | 1 |
| Myasthenia Gravis | 963 | CD53 | CD53 molecule | 1 |
| Myasthenia Gravis | 25819 | CCRN4L | CCR4 carbon catabolite repression 4-like (S. cerevisiae) | 1 |
| Myasthenia Gravis | 25897 | RNF19A | ring finger protein 19A, RBR E3 ubiquitin protein ligase | 1 |
| Myasthenia Gravis | 353 | APRT | adenine phosphoribosyltransferase | 1 |
| Myasthenia Gravis | 116 | ADCYAP1 | adenylate cyclase activating polypeptide 1 (pituitary) | 1 |
| Myasthenia Gravis | 285489 | DOK7 | docking protein 7 | 1 |
| Myasthenia Gravis | 406885 | MIRLET7C | microRNA let-7c | 1 |
| Myasthenia Gravis | 406937 | MIR145 | microRNA 145 | 1 |
| Myasthenia Gravis | 406947 | MIR155 | microRNA 155 | 1 |
| Myasthenia Gravis | 652070 | SCFV | single-chain Fv fragment | 1 |
| Myasthenia Gravis | 52 | ACP1 | acid phosphatase 1, soluble | 1 |
| Myasthenia Gravis | 128408 | BHLHE23 | basic helix-loop-helix family, member e23 | 1 |
| Myasthenia Gravis | 361 | AQP4 | aquaporin 4 | 1 |
| Myasthenia Gravis | 64221 | ROBO3 | roundabout, axon guidance receptor, homolog 3 (Drosophila) | 1 |
| Myasthenia Gravis | 26073 | POLDIP2 | polymerase (DNA-directed), delta interacting protein 2 | 1 |
| Myasthenia Gravis | 796 | CALCA | calcitonin-related polypeptide alpha | 1 |
| Myasthenia Gravis | 779 | CACNA1S | calcium channel, voltage-dependent, L type, alpha 1S subunit | 1 |
| Myasthenia Gravis | 717 | C2 | complement component 2 | 1 |
| Myasthenia Gravis | 29760 | BLNK | B-cell linker | 1 |
| Myasthenia Gravis | 51237 | MZB1 | marginal zone B and B1 cell-specific protein | 1 |
| Myasthenia Gravis | 57472 | CNOT6 | CCR4-NOT transcription complex, subunit 6 | 1 |
| Myasthenia Gravis | 64135 | IFIH1 | interferon induced with helicase C domain 1 | 1 |
| Myasthenia Gravis | 1.01E+08 | ERVW-4 | endogenous retrovirus group W, member 4 | 1 |
| Myasthenia Gravis | 7402 | UTRN | utrophin | 1 |
| Myasthenia Gravis | 3456 | IFNB1 | interferon, beta 1, fibroblast | 1 |
| Myasthenia Gravis | 3111 | HLA-DOA | major histocompatibility complex, class II, DO alpha | 1 |
| Myasthenia Gravis | 2952 | GSTT1 | glutathione S-transferase theta 1 | 1 |
| Myasthenia Gravis | 2694 | GIF | gastric intrinsic factor (vitamin B synthesis) | 1 |
| Myasthenia Gravis | 4656 | MYOG | myogenin (myogenic factor 4) | 1 |
| Myasthenia Gravis | 4684 | NCAM1 | neural cell adhesion molecule 1 | 1 |
| Myasthenia Gravis | 4914 | NTRK1 | neurotrophic tyrosine kinase, receptor, type 1 | 1 |
| Myasthenia Gravis | 5008 | OSM | oncostatin M | 1 |
| Myasthenia Gravis | 5066 | PAM | peptidylglycine alpha-amidating monooxygenase | 1 |
| Myasthenia Gravis | 3535 | IGL | immunoglobulin lambda locus | 1 |
| Myasthenia Gravis | 3976 | LIF | leukemia inhibitory factor | 1 |
| Myasthenia Gravis | 3120 | HLA-DQB2 | major histocompatibility complex, class II, DQ beta 2 | 1 |
| Myasthenia Gravis | 3447 | IFNA13 | interferon, alpha 13 | 1 |
| Myasthenia Gravis | 3575 | IL7R | interleukin 7 receptor | 1 |
| Myasthenia Gravis | 3439 | IFNA1 | interferon, alpha 1 | 1 |
| Myasthenia Gravis | 3438 | IFN1@ | interferon, type 1, cluster | 1 |
| Myasthenia Gravis | 3630 | INS | insulin | 1 |
| Myasthenia Gravis | 3135 | HLA-G | major histocompatibility complex, class I, G | 1 |
| Myasthenia Gravis | 969 | CD69 | CD69 molecule | 1 |
| Myasthenia Gravis | 5551 | PRF1 | perforin 1 (pore forming protein) | 1 |
| Myasthenia Gravis | 6607 | SMN2 | survival of motor neuron 2, centromeric | 1 |
| Myasthenia Gravis | 1435 | CSF1 | colony stimulating factor 1 (macrophage) | 1 |
| Myasthenia Gravis | 1398 | CRK | v-crk avian sarcoma virus CT10 oncogene homolog | 1 |
| Myasthenia Gravis | 7063 | THM | thymoma | 1 |
| Myasthenia Gravis | 7128 | TNFAIP3 | tumor necrosis factor, alpha-induced protein 3 | 1 |
| Myasthenia Gravis | 6606 | SMN1 | survival of motor neuron 1, telomeric | 1 |
| Myasthenia Gravis | 1490 | CTGF | connective tissue growth factor | 1 |
| Myasthenia Gravis | 6504 | SLAMF1 | signaling lymphocytic activation molecule family member 1 | 1 |
| Myasthenia Gravis | 5595 | MAPK3 | mitogen-activated protein kinase 3 | 1 |
| Myasthenia Gravis | 5894 | RAF1 | Raf-1 proto-oncogene, serine/threonine kinase | 1 |
| Myasthenia Gravis | 5920 | RARRES3 | retinoic acid receptor responder (tazarotene induced) 3 | 1 |
| Myasthenia Gravis | 1520 | CTSS | cathepsin S | 1 |
