# Supplementary material for: Building the drug-GO function network to screen significant candidate drugs for myasthenia gravis
Source: PLoS One. 2019 Apr 4;14(4):e0214857. doi: 10.1371/journal.pone.0214857 (PMC6448860; doi:10.1371/journal.pone.0214857)
Supplement: S4 Table — (DOC) [file pone.0214857.s006.doc]

Table S4. Target genes between 5 candidate drugs and GO functions.

| Drug | GO function | Gene |
| --- | --- | --- |
| Glucosamine | regulation of receptor activity | TNF,IFNG |
|  | defense response to virus | IFNG |
|  | response to drug | IFNG |
|  | cell surface receptor signaling pathway | IFNG |
|  | response to virus | TNF,IFNG |
|  | positive regulation of DNA binding transcription factor activity | TNF |
|  | cell cycle arrest | IFNG |
|  | positive regulation of smooth muscle cell proliferation | TNF |
|  | embryo implantation | MMP9 |
|  | collagen catabolic process | MMP9 |
|  | positive regulation of cell adhesion | TNF |
|  | negative regulation of smooth muscle cell proliferation | IFNG |
|  | positive regulation of protein complex assembly | TNF,IFNG |
|  | negative regulation of fibroblast proliferation | IFNG |
|  | endoplasmic reticulum unfolded protein response | IFNG |
|  | necroptotic signaling pathway | TNF |
|  | negative regulation of lipid storage | TNF |
|  | epithelial cell proliferation involved in salivary gland morphogenesis | TNF |
|  | positive regulation of protein localization to cell surface | TNF |
|  | regulation of the force of heart contraction | IFNG |
|  | negative regulation of myelination | IFNG |
|  | positive regulation of exosomal secretion | IFNG |
| Adalimumab | response to virus | TNF |
|  | positive regulation of DNA binding transcription factor activity | TNF |
|  | positive regulation of smooth muscle cell proliferation | TNF |
|  | positive regulation of cell adhesion | TNF |
|  | positive regulation of protein complex assembly | TNF |
|  | necroptotic signaling pathway | TNF |
|  | negative regulation of lipid storage | TNF |
|  | epithelial cell proliferation involved in salivary gland morphogenesis | TNF |
|  | positive regulation of protein localization to cell surface | TNF |
| Apremilast | regulation of receptor activity | TNF,IFNG,IL2 |
|  | positive regulation of cell proliferation | IFNG,IL2 |
|  | cell-cell signaling | IL2 |
|  | defense response to virus | IFNG |
|  | response to drug | IFNG |
|  | cell surface receptor signaling pathway | IFNG |
|  | response to virus | TNF,IFNG |
|  | positive regulation of DNA binding transcription factor activity | TNF |
|  | cell cycle arrest | IFNG |
|  | positive regulation of smooth muscle cell proliferation | TNF |
|  | positive regulation of tissue remodeling | IL2 |
|  | positive regulation of cell adhesion | TNF |
|  | negative regulation of smooth muscle cell proliferation | IFNG |
|  | positive regulation of protein complex assembly | TNF,IFNG |
|  | negative regulation of fibroblast proliferation | IFNG |
|  | positive regulation of cell growth | IL2 |
|  | endoplasmic reticulum unfolded protein response | IFNG |
|  | necroptotic signaling pathway | TNF |
|  | negative regulation of lipid storage | TNF |
|  | epithelial cell proliferation involved in salivary gland morphogenesis | TNF |
|  | positive regulation of protein localization to cell surface | TNF |
|  | regulation of the force of heart contraction | IFNG |
|  | negative regulation of myelination | IFNG |
|  | positive regulation of exosomal secretion | IFNG |
| Polaprezinc | regulation of receptor activity | TNF,IL6,NGF |
|  | cell-cell signaling | NGF |
|  | defense response to virus | IL6 |
|  | negative regulation of cell proliferation | IL6 |
|  | response to drug | IL6 |
|  | response to virus | TNF |
|  | positive regulation of DNA binding transcription factor activity | TNF,IL6 |
|  | positive regulation of epithelial cell proliferation | IL6 |
|  | positive regulation of smooth muscle cell proliferation | TNF,IL6 |
|  | platelet activation | IL6 |
|  | regulation of cell shape | IL6 |
|  | positive regulation of cell adhesion | TNF |
|  | response to heat | IL6 |
|  | positive regulation of protein complex assembly | TNF |
|  | response to cold | IL6 |
|  | response to antibiotic | IL6 |
|  | muscle cell cellular homeostasis | IL6 |
|  | negative regulation of hormone secretion | IL6 |
|  | response to electrical stimulus | IL6 |
|  | necroptotic signaling pathway | TNF |
|  | negative regulation of lipid storage | TNF,IL6 |
|  | endocrine pancreas development | IL6 |
|  | epithelial cell proliferation involved in salivary gland morphogenesis | TNF,IL6 |
|  | positive regulation of protein localization to cell surface | TNF |
|  | bone remodeling | IL6 |
|  | positive regulation of transmission of nerve impulse | IL6 |
|  | positive regulation of cell proliferation in bone marrow | IL6 |
| Etanercept | response to virus | TNF |
|  | response to hypoxia | LTA |
|  | positive regulation of DNA binding transcription factor activity | TNF |
|  | positive regulation of smooth muscle cell proliferation | TNF |
|  | positive regulation of cell adhesion | TNF |
|  | positive regulation of protein complex assembly | TNF |
|  | negative regulation of fibroblast proliferation | LTA |
|  | response to nutrient | LTA |
|  | necroptotic signaling pathway | TNF |
|  | negative regulation of lipid storage | TNF |
|  | epithelial cell proliferation involved in salivary gland morphogenesis | TNF |
|  | positive regulation of protein localization to cell surface | TNF |
|  | positive regulation of glial cell proliferation | LTA |
